# A comprehensive analysis on preservation patterns of gene co-expression networks during Alzheimer’s disease progression

**DOI:** 10.1186/s12859-017-1946-8

**Published:** 2017-12-20

**Authors:** Sumanta Ray, Sk Md Mosaddek Hossain, Lutfunnesa Khatun, Anirban Mukhopadhyay

**Affiliations:** 1 Department of Computer Science and Engineering, Aliah University, Kolkata, 700156 West Bengal India; 2 Department of Computer Science and Engineering, University of Kalyani, Kalyani, 741235 West Bengal India

**Keywords:** Module preservation measures, Gene co-expression networks, Hierarchical clustering, Rank aggregation

## Abstract

**Background:**

Alzheimer’s disease (AD) is a chronic neuro-degenerative disruption of the brain which involves in large scale transcriptomic variation. The disease does not impact every regions of the brain at the same time, instead it progresses slowly involving somewhat sequential interaction with different regions. Analysis of the expression patterns of the genes in different regions of the brain influenced in AD surely contribute for a enhanced comprehension of AD pathogenesis and shed light on the early characterization of the disease.

**Results:**

Here, we have proposed a framework to identify perturbation and preservation characteristics of gene expression patterns across six distinct regions of the brain (“EC”, “HIP”, “PC”, “MTG”, “SFG”, and “VCX”) affected in AD. Co-expression modules were discovered considering a couple of regions at once. These are then analyzed to know the preservation and perturbation characteristics. Different module preservation statistics and a rank aggregation mechanism have been adopted to detect the changes of expression patterns across brain regions. Gene ontology (GO) and pathway based analysis were also carried out to know the biological meaning of preserved and perturbed modules.

**Conclusions:**

In this article, we have extensively studied the preservation patterns of co-expressed modules in six distinct brain regions affected in AD. Some modules are emerged as the most preserved while some others are detected as perturbed between a pair of brain regions. Further investigation on the topological properties of preserved and non-preserved modules reveals a substantial association amongst “betweenness centrality” and ”degree” of the involved genes. Our findings may render a deeper realization of the preservation characteristics of gene expression patterns in discrete brain regions affected by AD.

**Electronic supplementary material:**

The online version of this article (doi:10.1186/s12859-017-1946-8) contains supplementary material, which is available to authorized users.

## Background

Alzheimer’s disease (AD) has been characterized as an irreversible, progressive neuro-degenerative incoherence in the brain and the major reason of dementia [1]. In AD, connections between cells in the brain are destroyed and eventually these cells die, which affects how the brain works. On its early onset, it is classified as short-term loss of memory. As the disease progresses, people suffers from issues with dialect, disorientation (letting in easily getting lost), loss of inspiration, mood swings, behavioral problems, not accomplishing self-care, and thus they are often kept isolated from family and the society. Its progression can be summarized in three stages: Early (“mild”), Middle (“moderate”) and Late (“severe”) [1, 2].

Typically, Alzheimer’s disease starts with very insignificant effects on the individuals capabilities or behavior. Initially it is characterized by memory loss, especially memory of more recent events which more often mistakenly classified as issues due to stress or mourning or in elderly persons, as the ordinary consequence of ageing (“mild stage”). As the disease advances (“moderate stage”), patient’s professional and social functioning continues to deteriorate because of increasing problems with memory, logic, speech, and initiative and the affected individual become incapable of performing natural activities of every day living [3]. In this stage, the most regions of the brain undergo severe impairment and drastically shrinks because of extensive cell death. During the final (“severe”) stage, patients become completely dependent upon caregivers [3, 4] and their dialect is lessened to basic expressions or many a time single words, finally prompting complete loss of discourse.

There are certain brain regions which are more susceptible to AD than others in terms of pathological and metabolic characteristics, although it does not affect all brain regions simultaneously [5–9]. It begins in the “entorhinal cortex” (EC) and “hippocampus” (HIP) [10]. Other brain regions such as the “middle temporal gyrus” (MTG) and the “posterior cingulate cortex” (PC) get affected later during progression of the disease [10, 11]. Thus, it is more significant to know the co-expression changes during the progression of AD from EC or HIP region to other brain regions. Dr. Alois Alzheimer characterized the symptoms of the disease in 1906. But the genesis of AD has continued to be elusive since then. Merely the “APOE” gene was observed to be related to AD in 1993. Thereafter, numerous analysis have been carried out to detect the genes which are expressed differentially in the Alzheimer’s disease influenced brain regions [12, 13]. In [14] Ray et al. differentiated 18-protein signatures in peripheral blood plasma which can be utilized to forecast the clinical syndromes of AD in advance well before the symptoms are apparent. Liang et al. [5] carried out a comprehensive analysis and discovered that “APOE”, “BACE1”, “FYN”, “GGA1”, “SORL1” and “STUB1 (CHIP)” genes are expressed differentially in postmortem gene expression dataset of six distinct brain regions. Moreover, they have indicated the genes which observed substantial changes in their expression patterns due to AD. Ray et al. [13] analyzed microarray data across four discrete brain regions (EC, HIP, PC, MTG) by constructing gene co-expression network for each region using differentially expressed genes amongst AD affected and normal control samples. They have identified the genes associated with “zero topological overlap” between a pair of regions specific networks to characterize the differences between the two brain regions.

A network-based systems biology methodology was proposed to analyze the Alzheimer’s disease associated pathways and their disfunctions among six discrete brain regions by Liu et al. in [15]. They have discovered the most pertinent AD associated pathways over the brain regions. Bertram et al. [16] executed an Alzheimer’s disease “genetic association” meta-analysis and discovered 20 polymorphisms in 13 genes which are strongly associated with AD. In [17], Puthiyedth et al. performed an comprehensive investigation with gene expression datasets of five distinct brain regions to get more insights into the mechanisms of AD. In this study they have discovered that “INFAR2” and “PTMA” were up-regulated whereas “FGF”, “GPHN”, “PSMD14” and “RAB2A” genes were down-regulated.

Langfelder et al. [18] established an unprecedented framework to unveil the relationship among the co-expressed modules using eigengene networks. To discover the resemblances and divergence within the network structures using co-expressed modules, considerable amount of computational mechanisms have been proposed [19–23]. To analyze the gene expression data of three different Hepatitis C related prognosis datasets, a biclustering based approach has been proposed in [24]. A novel computational approach has been introduced in [25] to discover the co-relation of gene expression levels in co-expressed modules among human blood and brain. Oldham et al. examined the evolutionary relationship within the chimpanzee and human brains using “gene co-expression networks” (GCN) in [19]. Hossain et al. unfolded the preservation affinity and changes of expression patterns in consensus (or shared) modules observed within distinct phases of evolvement in HIV-1 disease utilizing an eigengene network based approach [26].

This article presents a methodology to detect preservation pattern of gene co-expression network across six brain regions affected in AD. Here, we have adopted module preservation statistics introduced by Langfelder et al. [27] to detect the preserved patterns of gene expression. Initially, differentially expressed genes (DEGs) were extracted from the expression data of six different brain regions affected with AD. Next, we processed the data by taking common genes of a pair of regions at a time and built co-expression networks. Here, we have utilized the “Weighted Gene Co-Expression Network Analysis” (WGCNA) [28] framework to extract the co-expression modules from the networks. We have analyzed the preservation statistics of co-expression modules obtained from a pair of brain regions at a time. Moreover, we have employed a rank aggregation based method described in [29] to detect the overall changes of co-expression patterns among the brain regions in modular level. Here, we have used 12 measures to rank each co-expressed module and adopted a rank aggregation mechanism for combining those ranks. Every module gets an aggregated rank which describes its preservation characteristics in two brain regions. We have also identified “gene ontology” (GO) terms and the most significant KEGG pathways for the preserved and perturbed co-expressed modules corresponding to each pair of brain regions. Additionally, to investigate whether there exists any topological characteristics that distinguishes preserved module from non-preserved ones, we have analyzed the ‘degree’ and ‘betweenness centrality’ of all the proteins belonging to each preserved and non preserved module. In our present work, we have performed the whole analysis by taking EC and HIP regions as references and investigate the preservation patterns of gene expression inside other brain regions disrupted by AD.

## Methods

**Fig. 1:**
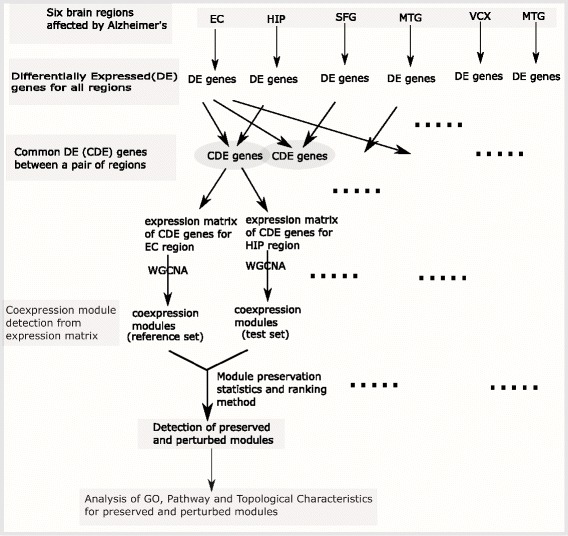
Schematic diagram describing the overall analysis carried out in the present article

This section describes our proposed framework for carrying out the present analysis. Figure 1 portrays the overall framework of this article. Initially, we have identified differentially expressed (DE) genes for all six brain regions and selected common DE genes between two regions at a time, as described in “Dataset preparation” section. Thereafter, for all the pairs of regions the common (or intersection) genes were used to construct co-expression modules using WGCNA framework mentioned in “Identification of gene co-expression modules” section. Next, we have employed the module preservation statistics introduced by Langfelder et al. in [27] to analyze the preservation and perturbation patterns of the identified co-expressed modules across a pair of regions [“Module preservation” section] and utilized a rank aggregation technique to rank the identified preserved and non-preserved modules [“Rank aggregation” section]. Moreover, we have identified the GO terms and the most significant KEGG pathways which are linked with the modules [“GO and pathway analysis of preserved and non-preserved modules” section]. Additionally, we have studied the topological characteristics of genes belonging to those modules in the “Topological insights into the preserved and perturbed modules” section.

### Dataset used

In this analysis we have used a publicly available microarray (“Affymetrix Human Genome U133 Plus 2.0”) expression dataset for six distinct brain regions (“EC”, “HIP”, “PC”, “MTG”, “SFG”, and “VCX”) which are either metabolically or histopathologically associated to Alzheimer’s disease [5]. Gene expression data was obtained from six functionally and anatomically discrete normal aged brain regions via laser capture microdissected neurons. The dataset is available in the “Gene Expression Omnibus” (GEO) with the series accession number “GSE5281”. Overall, the dataset contains 161 samples, among which 74 are normal or controls samples whereas 87 samples are affected by Alzheimer’s disease, with an average age of “79.8 ± 9.1” years. Each sample consists of 54675 genes. The samples were obtained from “clinically” and “neuro-pathologically” categorized Alzheimer’s impacted persons at three distinct AD centers (having an average post-mortem interval (PMI) of 2.5 h). We have used the data collected from “entorhinal cortex” [EC; “Brodmann area (BA) 28 and 34”], “hippocampus” [HIP; “CA1 region”], “posterior cingulate cortex” [PC; “BA 23 and 31”], “medial temporal gyrus” [MTG; “BA 21 and 37”], “superior frontal gyrus” [SFG; “BA 10 and 11”], and “primary visual cortex” [VCX; “BA 17”]. AD involved samples were associated with a Braak stage varying from III to VI [10, 30]. Expression data for every sample was acquired from roughly around 500 number of pyramidal neurons. Entire dataset is comprised of AD affected and control samples of six distinct brain regions. These are EC region (10 AD and 13 control), HIP region (10 AD and 13 control), MTG region (16 AD and 12 control), PC region (9 AD and 13 control), SFG region (23 AD and 11 control) and VCX region (19 AD and 12 control).

### Dataset preparation

First of all, as a preprocessing step, we have performed *l* *o* *g*_2_ transformation of the gene expression data in order to have equivalent effect on the two-fold increase or decrease in gene expression data in *log*-scale. Then, the gene expression data is normalized with the help of ‘manorm()’ Matlab function to eliminate the inconstancies in microarray experimentation that influenced the observed gene expressions as a consequence of deviation in the experimental process, experimenter biasness, samples acquisition-processing or additional machine specifications. The manorm() function scales the values in each sample (column) of the gene expression matrix with dividing them by the mean sample intensity.

**Fig. 2:**
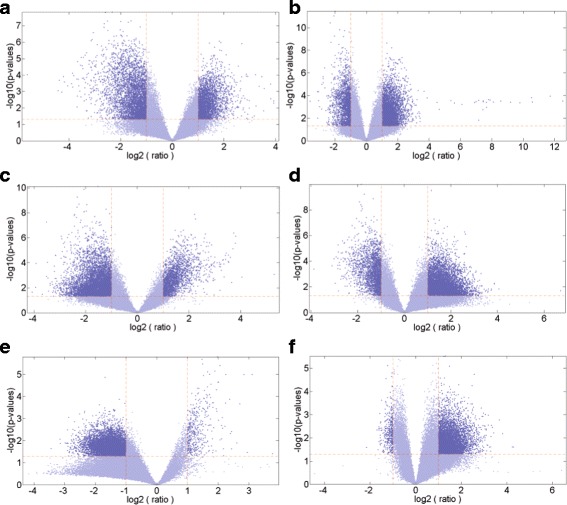
Volcano plots of gene expressions of control and affected samples corresponding to all six brain regions in AD. Panel (**a**) EC (**b**) HIP (**c**) MTG (**d**) PC (**e**) SFG (**f**) VCX. In each volcano plot, a scatter plot is shown plotting significance (− log10 (*p*-*value*)) versus fold change of gene expression ratio (log2(*r* *a* *t* *i* *o*)) of microarray data

Next, to evaluate the differential expression of genes, we processed the datasets of all six brain regions using a standard two-tailed and two-sample t-test taking control and affected samples of a single region at a time. For discovering the patterns how gene expressions are mutated within control and affected samples, six volcano plots were generated, one per brain region [Fig. 2]. We have employed “two samples t-test” for detecting differential expression of genes and the statistical significance was measured through *p*-value. Corresponding to every brain region fold changes for expression value of every gene within control and affected samples was also computed. The cut off threshold at significance level of 0.05 (indicated with ‘horizontal red dashed’ lines) and fold change at 2 (indicated with ‘vertical red dashed’ lines) was set. The plots shown in Fig. 2 indicates the genes which are expressed differentially among control and affected samples for all brain regions at the chosen level of significance. Table 1 dictates the count of the selected DEGs for the six distinct brain regions.

**Table 1:** Number of differentially expressed (DE) genes in the six brain regions

Sl No.	Region	No. of DE genes
1	EC	12629
2	HIP	13534
3	MTG	14090
4	PC	17712
5	SFG	11963
6	VCX	14126

Following the identification of six sets of DEGs, one for each brain region, the mutual DEGs within a pair of regions was computed at a time. The numbers of common DEGs among the six brain regions while considering EC and HIP regions as reference datasets are shown in Table 2.

**Table 2:** Number of differentially expressed common genes (intersection genes) among the six brain regions taking two regions of interest at a time. Here, we have chosen EC and HIP region as reference datasets

Sl No.	Regions compared	No. of intersection genes
1	EC-HIP	4083
2	EC-MTG	4175
3	EC-PC	4527
4	EC-SFG	3288
5	EC-VCX	3325
6	HIP-MTG	5204
7	HIP-PC	7156
8	HIP-SFG	4719
9	HIP-VCX	4216

The common genes (or ‘intersection genes’) were utilized for constructing a pair of gene co-expression networks, each of which corresponds to one region. For producing gene co-expression networks and detecting modules the popular WGCNA framework [28] have been availed here.

### Identification of gene co-expression modules

In the present section, we have described the step by step procedure for constructing gene co-expression modules for our present work.

#### Constructing gene co-expression networks through adjacency matrix

Network may easily be expressed using an “adjacency matrix” *A* *d* *j*=[*M*_*uv*_] that reflects the levels of interconnectedness of nodes within themselves. With a symmetric adjacency matrix comprising of [*m*×*m*] components a gene co-expression network (GCN) can be constructed in which every node represents a gene [31].

To represent an unweighted network, we assign a weight 1 if a pair of nodes *u* and *v* are connected (adjacent) to each other, or a value 0 if nodes are not adjacent to each other to every individual element *M*_*uv*_ in the adjacency matrix. For a weighted network, the intensity level of connection among the nodes *u* and *v* is denoted by 0≤*M*_*uv*_≤1.

1 $$\begin{array}{*{20}l} 0 \leq M_{uv} \leq 1,\\ M_{uv} = M_{vu}, \\ M_{uu}=1. \end{array} $$

For notational convenience, we have utilized the *“vectorizeMatrix()”* function of the WGCNA package [28] which accepts a symmetric matrix *A* *d* *j*∈*R* ^*m*×*m*^ and a vector consisting of *m*(*m*−1)/2 non-redundant elements is returned as output [27].

2 $$ \begin{aligned} {}&vectorizeMatrix(Adj)=\\& \left\{M_{21}, M_{31}, M_{32}, M_{41}, M_{42}, M_{43}, \ldots, M_{mm-1}\right\}. \end{aligned}  $$

Here, for each pair of regions two separate GCNs were created by calculating the ‘Spearman correlation’ between expression profiles of intersection genes. Thus, we construct ten pairs of co-expression networks, among them 5 pairs are built by taking EC region as reference and other 5 pairs are constructed by taking HIP region as reference.

#### Scale free network transformation

We have adopted the “scale free” transformation principles introduced by Zhang et al. [28] to give emphasis upon the high adjacency values sacrificing insignificant ones and to fulfill the “scale free topology” criteria. Thus the correlation coefficients for the entire gene co-expression matrix were elevated to a constant power *λ*.

3 $$ {Power}_{uv}(Adj, \lambda) = M^{\lambda}_{uv}.  $$

**Fig. 3:**
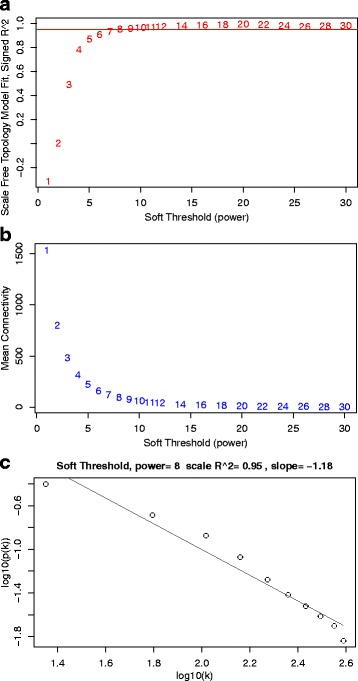
Scale free transformation plots for EC region gene co-expression network using differentially expressed intersection genes with HIP region. The plots shows the network properties of gene co-expression network of EC region for different soft thresholds. For different soft thresholds, the plots visualize the scale free topology fitting index (panel -**a**), the mean connectivity (panel -**b**). Panel **c** shows the scale free topology plot of the EC region co-expression network that is constructed with the power adjacency function power (*λ*=8). This scatter plot between *l* *o* *g*_10_(*p*(*k*)) and *l* *o* *g*_10_(*k*) shows that the network satisfies a scale free topology approximately (a straight line is indicative of scale-free topology)

We have discovered that the gene expression dataset of intersection genes of the EC region (when compared to HIP region) conforms to the “scale free topology” criterion roughly at soft threshold power *λ*=8 since the “scale-free topology model fitting index”: *R* ^2^, attains a high thresholds value (0.95) [Fig. 3 a and b]. Thereafter, utilizing *λ* as an argument we have executed the “softConnectivity()” function of the WGCNA package to compute the connectivities among the intersection genes and drawn the scale free plot [Fig. 3 c]. Let *p*(*k*) be the probability of the nodes with connectivity *k*. A linear association among *l* *o* *g*(*p*(*k*)) and *l* *o* *g*(*k*) has been noticed in Fig. 3 c which further affirms that scale free transformation of the EC gene co-expression networks attains approximately at *λ*=8.

Similarly, we have utilized the procedure described above to convert all other gene co-expression networks into scale free networks.

#### Topological overlap matrix based similarity-dissimilarity measures

In network analysis field a primary goal is the discovery of the modules or groups of strongly correlated genes. It can be achieved by inspecting the resemblance in connection intensities or significant “topological overlap” within the genes. In this article, for discovering modules in the GCNs, we have utilized the “Topological Overlap Matrix” (TOM) similarity measure [32–34] that represents the extent of similarity between a pair of genes in respect of commonality among the genes they are associated with.

TOM is represented as

4 $${} {TOM}_{uv}(Adj)=\frac{\sum_{z \neq u,v}M_{uz}M_{zv}+ M_{uv}}{min(\sum_{z \neq u}M_{uz},\sum_{z\neq v}M_{zv}) +1- M_{uv}}.  $$

TOM dissimilarity matrix may readily be obtained by employing the expression indicated below:

5 $$\begin{array}{@{}rcl@{}} D_{uv}&=& {Dissim}_{uv}(TOM(Adj)) \\&=& 1-{TOM}_{uv}(Adj). \end{array} $$

#### Module discovery through hierarchical clustering

In this article, we have discovered the co-expressed network modules with the application of average linkage hierarchical clustering. Here we have applied the “dynamic tree cut” algorithm [35] by utilizing the pairwise node dissimilarity *D*_*uv*_ as input argument and the resultant stems on the dendrogram are marked as modules.

### Module preservation

In the present article, we have exerted the module preservation statistics introduced by Langfelder et al. in [27] to discover the preservation and perturbation patterns of the identified co-expressed modules across a pair of independent networks. We have adopted 12 preservation statistics to investigate whether an identified module presents in a “reference network” (having adjacency matrix *A* *d* *j* ^[*r*]^) may be observed within an independent disjoint “test network” (having adjacency *A* *d* *j* ^[*t*]^). Based on the values of each of the preservation measures, all the identified modules in the reference network were assigned 12 different ranks.

Table 3 presents the list of module preservation statistics we have utilized in our present work to discover a module that exist in a given network may be detected within a completely uncorrelated network and to rank the identified modules based on those measures. In section [“Module preservation measures”], we have briefly described about those measures.

**Table 3:** List of the preservation measures utilized to rank identified modules

Sl No.	Preservation measures	Type
1	meanAdj	Density
2	meanMAR	Density
3	medianRankDensity	Density
4	propVarExplained	Density
5	corr.kIM	Connectivity
6	corr.kME	Connectivity
7	corr.kMEall	Connectivity
8	corr.corr	Connectivity
9	corr.MAR	Connectivity
10	medianRankConnectivity	Connectivity
11	meanKME	Density + Connectivity
12	meanCorr	Density + Connectivity

The ranking measures adopted here are associated with various density, connectivity and eigengene based statistics which are elongation of different fundamental measures that operates on nodes. We have utlized the following fundamental measures: Density, Maximum Adjacency Ratio, Module Membership (kME), Clustering Coefficient and Intramodular Connectivity (kIM).

- Density [31, 36]: Module density within a network represents the average connection (association) strengths among every couple of nodes in that module. Here, the connection strength is defined as the correlation coefficient among the expression profiles of every couple of genes (or nodes) within that module. Thus, the density of a module represents the mean adjacency and is expressed as:
  6 $$ density^{(p)} = mean(vectorizeMatrix(Adj^{(p)})),  $$
  where *A* *d* *j* ^(*p*)^ represents the adjacency matrix for all nodes present within the module *p*. Intuitively, higher module-density indicates a module with strongly interconnected nodes.
- Maximum Adjacency Ratio (MAR) [36]: With reference to a weighted network the MAR of a node *u* is expressed as
  7 $$ {MAR}_{u}=\frac{\sum_{u \ne v} w(u,v)^{2}}{\sum_{u \ne v} w(u,v)},  $$
  where *w*(*u*,*v*) corresponds to the connection strength associated with the nodes *u* and *v*.
  MAR is characterized exclusively for weighted networks, since it is constant (=1) in an unweighted network. The MAR statistics can easily employed in connection with a module by computing the average MAR score of every node present in the module.
  To compare the MAR scores among two independent networks, we have computed the mean MAR scores of all the modules of those two networks and obtained their correlation scores (corr.MAR). The MAR measure may also be exploited for discovering whether a hub gene accomplishes mild associations with a large number of genes or apparently firm associations with comparatively small number of genes.
- Module Membership (kME) [27]: There exists a plenty of module discovery techniques that results in co-expressed network modules comprising of significantly correlated nodes. Such modules can be summarized with the first principal component of the associated module expression matrix which is designated as the module eigengene (ME) [18]. Module Membership (kME) of a gene (or node) *u* with respect to module *p* represents the correlation among the expression profile of the node and the expression profile of the module eigengene. In an abstract view it specifies how adjacent the node *u* is to the module *p* and its values ranges within [−1,1].
  8 $$ kME^{p}_{u}=corr({expr}_{u},ME^{p}),  $$
  where, *e* *x* *p* *r*_*u*_ denotes the expression profile of gene (or node) *u* and *M* *E* ^*p*^ represents the module eigengene for the module *p*.
- Clustering Coefficient [28]: Within a network the clustering coefficient of a node is a measure of the degree of interconnectedness with its adjacent nodes. Let *e*_*u*_ be the total number of direct links (edges) with the nodes associated with node *u* and *n*_*u*_ be the number of nodes directly connected to node *u*. Then the clustering coefficient (CC) for a node *u* is computed as:
  9 $$ {CC}_{u}=\frac{2e_{u}}{n_{u}(n_{u}-1)}.  $$
  By definition, the clustering coefficient of a node ranges from 0 to 1. The average clustering coefficient can be utilized to assess whether the network exhibits a modular organization [32]. Among numerous alternatives available, in this article we have utilized the weighted generalization of clustering coefficient for co-expression network established in [28].
  Here the CC measure quantifies the magnitude of connection strength observed in the neighborhood of a node (*u*) and expressed as:
  10 $$ {CCW}_{u}=\frac{\sum_{v \ne u} \sum_{z \ne v, u} w(u,v)w(v,z)w(z,u)}{(\sum_{v \ne u} w(u,v))^{2}-\sum_{v \ne u} w(u,v)^{2} },  $$
  where *w*(*p*,*q*) is the weight of each edge coming out from node *p*. Here, the connection strength of the edges (weights) are normalized to the highest weight in the network. Average clustering coefficient of a module within a network has been computed by finding the mean weighted clustering coefficient of all nodes in that module.
- Intramodular Connectivity (kIM) [27]: The intramodular connectivity of a node represents the sum of connection strengths of that node to every other nodes in a specified module. Thus if a node is strongly connected with all other nodes in a module then it has a high intramodular connectivity. In this article, we have utilized this measure to obtain the similarity scores for alikeness of hub nodes within two independent networks.
  The intramodular connectivity for a node *u* in a module *p* is defined as
  11 $$ kIM^{p}_{u}= \sum_{v \in \mathcal{M}_{p},v \ne u} w(u,v)^{p}.  $$

### Module preservation measures

Following is the brief description about the 12 different preservation measures that have been employed in our present work.

1. meanAdj: meanAdj for a module provides the density of that module. Intuitively, a module *p* in a reference network is said to be conserved provided the module has a satisfactory density (adjacency) inside the test network. It is expressed as:
  12 $$ meanAdj = mean(vectorizeMatrix(Adj^{p})).  $$
2. meanMAR: meanMAR of a module provides the mean of the maximum adjacency ratios (MARs) of every node (*u*) inside the module (*p*) and is expressed as:
  13 $$\begin{array}{*{20}l} mean\left({MAR}_{u}^{^{p}}\right),\\ \text{where}, {MAR}_{u}=\frac{\sum_{u \ne v} w(u,v)^{2}}{\sum_{u \ne v} w(u,v)}. \end{array} $$
3. medianRankDensity: This represents the median rank of a module *p* based on all density statistics measures. It is expressed as:
  14 $$ {}medianRankDensity= {median}_{a \epsilon DensityStatistics} rank^{p}_{a},  $$
  where, $rank^{p}_{a}$ represents rank of a module *p* based on a density statistics measure *a*.
4. propVarExplained: propVarExplained (‘proportion of variance explained’) is computed by finding the mean from the square of the module membership (kME) scores of every nodes inside a module (*p*). It is expressed as:
  15 $$ propVarExplained = {mean}_{u \in \mathcal{M}_{p}}{\left({kME}_{u}^{[t](p)}\right)^{2}},  $$
  where, ${kME}_{u}^{[t](p)}$ indicates module membership score of node *u* in the module *p* in the network *t*.
5. corr.kIM: It represents the association among intramodular connectivities of every nodes inside a module between a pair of networks. It is expressed by:
  16 $$ corr.kIM = corr(kIM^{[r](p)},kIM^{[t](p)}),  $$
  where, *k* *I* *M* ^[*k*](*p*)^ represents the intramodular connectivity of module *p* in network *k*.
6. corr.kME: corr.kME for a module indicates the association among the module membership (kME) scores of every node inside the module between a pair of networks. It is expressed as:
  17 $$ corr.kME = {corr}_{u \in \mathcal{M}_{p}}\left({kME}_{u}^{[r](p)},{kME}_{u}^{[t](p)}\right),  $$
  where, ${kME}_{u}^{[k](p)}$ represents the module membership of node *u* in the module *p* in network *k*.
7. corr.kMEall: corr.kMEall of a module, signifies the association among the module membership (kME) scores of every nodes between a pair of networks. It is expressed as:
  18 $$ corr.kMEall = corr({kME}_{u}^{[r](p)},{kME}_{u}^{[t](p)}),  $$
  where, ${kME}_{u}^{[k](p)}$ indicates the module membership score of a node *u* inside the module *p* in network *k*.
8. corr.corr: It represents the correlation between connectivity patterns inside a module (*p*) among two networks. It is expressed as:
  19 $$ \begin{aligned} {}corr.corr^{(p)} &= corr\left(vectorizeMatrix(C^{[r](p)}\right),\\&vectorizeMatrix(C^{[t](p)})), \end{aligned}  $$
  where, *C* ^[*k*](*p*)^ represents the correlation matrix (*C*=[*c*_*uv*_]) for all pair of nodes (*u*, *v*) within the module *p* in the network *k* whose elements are expressed as:
  20 $$ c_{uv} = corr({expr}_{u},{expr}_{v}).  $$
9. corr.MAR: It signifies the association among maximum adjacency ratios (MARs) of every node inside a module among a pair of networks. It is expressed as:
  21 $$ corr.MAR^{(p)} = corr(MAR^{[r](p)},MAR^{[t](p)}),  $$
  where, *M* *A* *R* ^[*k*](*p*)^ indicates the maximum adjacency ratio (MAR) of the module *p* in the network *k*.
10. medianRankConnectivity: This represents the median rank of a module *p* based on all connectivity statistics measures. It is expressed as:
  22 $$ \begin{aligned} {}medianRank&Connectivity^{(p)} \\&= {median}_{a \epsilon ConnectivityStatistics} rank^{p}_{a}, \end{aligned}  $$
  where, $rank^{p}_{a}$ represents rank of a module *p* based on a connectivity statistics measure *a*.
11. meanKME (or meanSignAwareKME): Mean sign-aware module membership (meanKME) of a module *p* within a test network (*t*) is determined by computing the average of the module membership (kME) scores of all nodes in the module inside the test network multiplied by the corresponding score on the reference network. It can be expressed by:
  23 $$ {{\begin{aligned} meanKME^{[t](p)} = {mean}_{u \in \mathcal{M}_{p}}\left\{sign\left({kME}_{u}^{[r](p)}\right){kME}_{u}^{[t](p)}\right\}, \end{aligned}}}  $$
  where, ${kME}_{u}^{[k](p)}$ indicates the module membership (kME) score of the node *u* within the module *p* in the network *k*.
12. meanCorr (or meanSignAwareCorrDat): Mean sign-aware correlation of a module *p* within a test network (*t*) is defined as the average correlation values of every pair of nodes in that test network multiplied by sign of the corresponding scores on the reference network. It is expressed as:
  24 $$ {{\begin{aligned} meanCorr^{[t](p)} \!= \!mean \left\{vectorizeMatrix\left(sign\left(c_{uv}^{[r](p)}\right)c_{uv}^{[t](p)} \!\right) \!\right\}, \end{aligned}}}  $$
  where, $c_{uv}^{[k](p)}$ indicates the correlation score among the expression profiles of genes (or nodes) *u* and *v* inside the module *p* in the network *k* which has been expressed in the Eq. [20].

### Evaluating significance of observed statistics

The outcomes of the module preservation measures are generally dependent on several factors like the size of the network, size of the modules, number of measurements, etc. Hence, to assess whether a preservation statistics is significant or not, we have performed permutation tests. The module labels were randomly permuted in the test network and results of preservation statistics were obtained repeatedly for thirty times. Then, we have computed the mean (*μ*_*i*_) and standard deviation (*σ*_*i*_) of the permuted values for each statistics (*i*) and approximation of that statistics (*Z*_*i*_) was obtained [27]:

25 $$ Z_{i} = \frac{{Obs}_{i} - \mu_{i}}{\sigma_{i}}  $$

where, *O* *b* *s*_*i*_ denotes the observed value for the statistics *i*.

Moreover, all of the density and connectivity based preservation measures were summarized using three composite Z statistics *Z*_*density*_, *Z*_*connectivity*_ and *Z*_*summary*_ as given below [27]:

26 $$ {{\begin{aligned} &{}Z_{density} = median(Z_{meanCorr}, Z_{meanAdj}, Z_{propVarExpl}, Z_{meankME}). \end{aligned}}}  $$

27 $$\begin{array}{*{20}l} &{}Z_{connectivity} = median(Z_{corr.kIM}, Z_{corr.kME}, Z_{corr.corr}). \end{array} $$

28 $$\begin{array}{*{20}l} &{}Z_{summary} = \frac{Z_{density} + Z_{connectivity}}{2}. \end{array} $$

### Rank aggregation

Based on the values of the 12 preservation measures listed in Table 3, all the identified modules in the reference network were assigned 12 different ranks which signifies their preservation patterns in comparison to a test network.

Then, we have employed the rank aggregation technique proposed in [29] to obtain an optimum consolidated rank for each of the identified modules. This weighted rank aggregation method utilizes Monte Carlo cross-entropy approach that optimizes a distance criterion to combine the 12 different ranks of an identified co-expressed preserved module in a reference network based on 12 different preservation measures.

Low ranks of a module signify that the module is highly preserved inside the test network whereas high rank indicates its preservation characteristics is low in the test network.

## Results and discussion

This section provides the outcomes of our analysis to reveal the intramodular and topological changes in the modular architecture in each pair of brain regions perturbed with Alzheimer’s disease.

### Identification of co-expressed modules

**Fig. 4:**
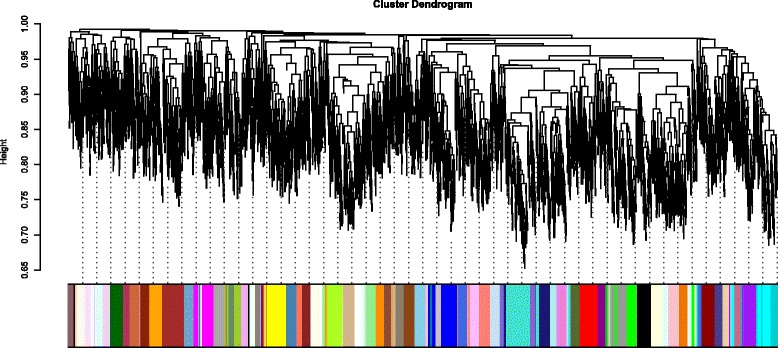
Hierarchical clustering dendrogram for gene co-expression network of EC brain region using the differentially expressed intersection genes with HIP region

We have identified co-expressed modules within the gene co-expression networks for each brain region using gene expression data of differentially expressed intersection genes with all other brain regions. Here, we have employed the dissimilarity measure expressed in [Eq. 4] with average linkage hierarchical clustering algorithm to detect such co-expressed modules. All the genes within the identified modules have been assigned same color code. Minimum module size we have considered in this work is 30. The genes those are allotted to none of the co-expressed modules are labelled in grey color. Figure 4 shows the hierarchical clustering dendrogram for gene co-expression network of EC brain region using the differentially expressed intersection genes with HIP region. From Table 4, it can be observed that the ‘brown’ module consists of 134 genes and it is associated with the GO term “microtubule cytoskeleton organization” (*p*-Value of 0.0092) and “Sphingolipid signaling” KEGG pathway (*p*-Value = 0.008). It is established in different literatures that cytoskeleton is progressively disrupted in the Alzheimer’s disease [37, 38]. Major component of cytoskeleton is microtubules which is regarded as critical structure for neuronal morphology. In AD affected neurons breakdown of microtubules is also an well established phenomenon [38].

**Table 4:** Significant GO Terms and KEGG Pathways for EC-HIP regions pair

Sl No	Module name	Aggregated rank	Module size	GO term	GO ID	*p*-Value	Pathway	*p*-Value
1	Antiquewhite4	9	32	Positive regulation of organ growth	GO:0046622	1.60E-02	Oxidative phosphorylation	2.50E-02
2	Bisque4	10	47	Phosphatidic acid biosynthetic process	GO:0006654	5.50E-02	Fat digestion and absorption	6.60E-02
3	Black	3	97	Membrane depolarization	GO:0051899	5.10E-03	Estrogen signaling pathway	1.00E-03
4	Blue	8	134	Protein stabilization	GO:0050821	8.70E-03	Oxytocin signaling pathway	5.70E-04
5	Brown	4	134	Microtubule cytoskeleton organization	GO:0000226	9.20E-03	Sphingolipid signaling pathway	8.00E-03
6	Darkmagenta	5	57	Positive regulation of GTPase activity	GO:0043547	9.10E-04	Spliceosome	7.20E-02
7	Darkolivegreen	7	58	Negative regulation of mitochondrial membrane potential	GO:0010917	8.30E-03	Not found	NA
8	Ivory	2	52	Cell migration	GO:0016477	5.20E-02	Not found	NA
9	Lightsteelblue1	1	54	Mucosal immune response	GO:0002385	1.00E-02	Measles	6.10E-02
10	Royalblue	6	77	Transport	GO:0006810	3.40E-03	Not found	NA
11	Brown4	55	48	Positive regulation of apoptotic process	GO:0043065	2.30E-02	Not found	NA
12	Darkgreen	58	74	Negative regulation of transcription: DNA-templated	GO:0045892	5.00E-02	Not found	NA
13	Honeydew1	56	32	Termination of RNA polymerase II transcription	GO:0006369	7.70E-02	Not found	NA
14	Lavenderblush3	61	32	Mitochondrial ATP synthesis coupled proton transport	GO:0042776	2.70E-02	Gastric acid secretion	8.10E-02
15	Lightpink4	53	36	Skeletal muscle acetylcholine-gated channel clustering	GO:0071340	1.30E-02	Fatty acid degradation	8.60E-02
16	Salmon4	60	41	Sphingolipid metabolic process	GO:0006665	2.70E-02	Proximal tubule bicarbonate reclamation	6.40E-02
17	Sienna3	54	57	Adult walking behavior	GO:0007628	7.80E-02	Not found	NA
18	Skyblue	52	63	Protein transport	GO:0015031	1.10E-04	Not found	NA
19	Thistle2	57	43	Regulation of autophagosome assembly	GO:2000785	2.80E-02	Not found	NA
20	Yellowgreen	59	56	Positive regulation of apoptotic process	GO:0043065	4.40E-03	Insulin resistance	6.40E-03

Sphingolipids play an important roles in signal transduction. In [39], it is reported that the perturbation of “sphingomyelin metabolism” is the main event in neurons degeneration that occurs in AD. Similarly, the ‘black’ module contains of 97 genes and it is associated with the GO term “membrane depolarization” (*p*-Value of 0.0051) and “Estrogen signaling” KEGG pathway (*p*-Value = 0.001). By and large, most of the identified modules are significantly enriched with known and relevant gene ontology terms and associated with KEGG pathways.

### Preserved modules in each pair of regions

**Fig. 5:**
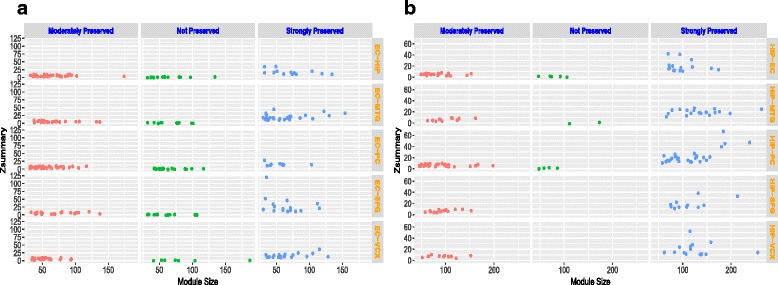
Figure shows plots of *Z*_*summary*_ with module size of co-expression modules for each pair of brain region. **a** EC region as reference data. **b** HIP region as reference data. First column shows the modules having *Z*_*summary*_ value within 2 to 10, while second third columns shows the scatter plot of modules having *Z*_*summary*_ values less than 2 and greater than 10 respectively

After obtaining module preservation statistics for each module, we have analyzed the preservation and perturbation structure of co-expression pattern of these modules. In particular, we have assumed coexpression network resulting from EC or HIP regions as reference dataset and the co-expression network of other regions as test datasets. For example, at a time we have computed the preservation statistics of co-expression modules belonging to one among the EC or HIP regions as reference dataset while the modules of one of the rest five other regions as test dataset. The aim is to study the preservation pattern of co-expression modules of EC and HIP regions in other affected brain regions. So, we have computed the preservation statistics of the co-expression modules for the following pair of regions, EC-HIP, EC-PC, EC-SFG, EC-VCX and EC-MTG by taking EC region as reference and HIP-EC, HIP-PC, HIP-SFG, HIP-VCX and HIP-MTG by taking HIP region as reference. In Fig. 5 a and b, we have shown the *Z*_*summary*_ values of all the co-expression modules with module size for EC and HIP regions, respectively. Each row of the Fig. 5 represents scatter plot of *Z*_*summary*_ values with the module size for each pair of regions. Following the convention of [27] the value of *Z*_*summary*_ higher than ten or less than two generally represent preserved modules or non-preserved module, respectively, whereas the value within 2 to 10 represents moderately preserved module. We have displayed the *Z*_*summary*_ values with module size in three columns in Fig. 5. Column 1 represents moderately preserved module, while column 2 and column 3 represent non-preserved and preserved modules of each region pair by considering EC as reference dataset. It emerges from the analysis that the number of strongly preserved module for EC-MTG region (26 out of 64 : 40%) is more than the other pair of regions (for EC-HIP: 13 out of 62 : 21%, EC-PC : 10 out of 79 : 12.65%, EC-SFG : 16 out of 49 :32.65%, and EC-VCX: 20 out of 52 : 38.46%)). For co-expression modules of HIP region, it can also be seen that for HIP-MTG region number of strongly preserved module is higher (19 out of 31) than the other pair of regions: for HIP-EC : 15 out of 40, for HIP-PC : 28 out of 60 for HIP-SFG : 11 out of 24, and for HIP-VCX : 15 out of 25.

**Fig. 6:**
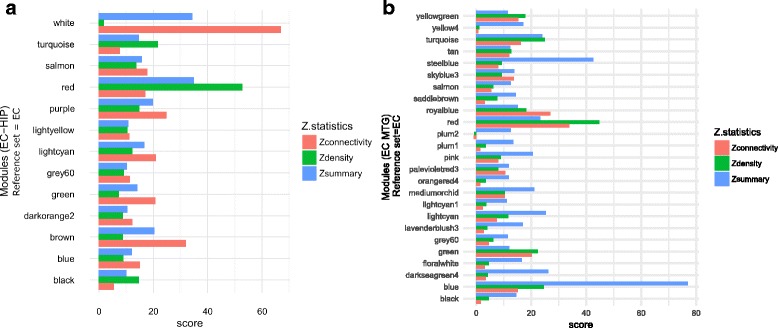
Bar plot showing *Z*_*density*_, *Z*_*connectivity*_ and *Z*_*summary*_ values of co-expression modules having *Z*_*summary*_ greater than ten. Panel (**a**) shows the results for modules in HIP region and panel (**b**) shows the same for MTG region. Both results are calculated by taking EC region as reference

For more detail investigation, we have generated a bar diagram in the Fig. 6 showing the values of *Z*_*summary*_, *Z*_*connectivity*_ and *Z*_*density*_ of preserved modules (*Z*_*summary*_ value ≥ 10) of HIP and MTG region taking EC region as reference. It can be seen from the Fig. 6 a that ‘white’ and ‘red’ module have higher *Z*_*summary*_ value thereby treated as the most preserved module between two regions EC and HIP. For MTG region 25 modules have *Z*_*summary*_ value more than 10. Figure 6 b shows the bar plot for MTG region taking EC as reference region. It can be seen from the Fig. 6 b that module ‘blue’ and ‘steelblue’ achieve *Z*_*summary*_ value higher than other. The *Z*_*summary*_, *Z*_*connectivity*_ and *Z*_*density*_ of preserved modules (*Z*_*summary*_ value 10) for HIP-EC and HIP-MTG regions pairs are provided in Additional file 1: Figure S1.

We have also compared the module preservation statistic *MedianRank* [27] of all co-expressed modules for each pair of regions taking EC and HIP as references. Z statistics generally depends on the module size, and in our case, the obtained co-expression modules are of different size, so it is better to focus on composite preservation statistics *MedianRank* which is defined as follows:

29 $$ {{}\begin{aligned} &MedianRank = \\ &\quad\frac{medianRankDensity + medianRankConnectivity}{2}. \end{aligned}}  $$

**Fig. 7:**
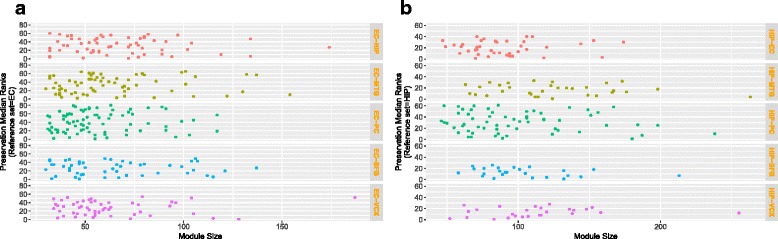
Figure shows plots of *MedianRank* values with module size of co-expression modules for each pair of brain region. **a** EC region as reference data. **b** HIP region as reference data. Each row in the figure corresponds to five other regions while taking either EC or HIP region as reference at a time

**Fig. 8:**
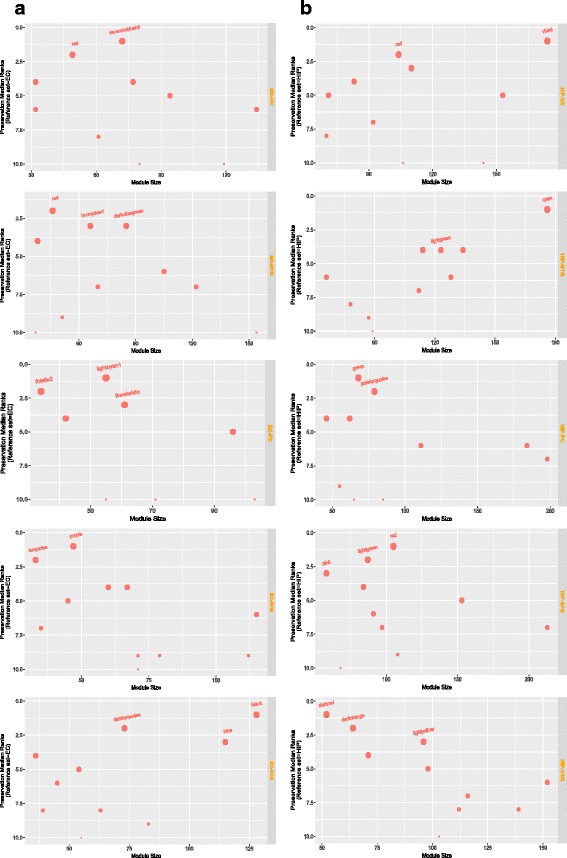
Figure shows scatter plots of *MedianRank* vs module size of co-expressed modules having *MedianRank* value less than 10. **a** EC region as reference data. **b** HIP region as reference data. Each panel shows the scatter plot of the modules identified in five other brain regions taking either EC and HIP regions as reference at a time. Here the modules with lower *MedianRank* are indicated with bigger filled circles

In Fig. 7, we have shown a scatter plot for the *MedianRank* values of all the modules obtained from each pair of regions by taking EC and HIP regions as reference datasets. From this figure one can see that for regions pair EC-SFG, *MedianRanks* of modules are lower than other pairs of regions. Number of modules having *MedianRank* less than 10, taking EC region as reference is as follows: for EC-HIP 10 out of 61 modules (16.4%), for EC-PC 8 out of 80 (10%), for EC-VCX 10 out of 53 (18.88%), for EC-SFG 11 out of 50 (22%) and for EC-MTG 10 out of 65 (15.38%). Number of modules having *MedianRank* less than 10 taking HIP region as follows: for HIP-EC 10 out 0f 40, for HIP-PC 9 out of 60, for HIP-SFG 9 out of 24, HIP-VCX 9 out of 25, and for HIP-MTG 9 out of 31. As low value of *MedianRank* represents preserved module, so it is observed from the figure that the most of the co-expression modules of EC region are more preserved in SFG than other regions, while very few of them are preserved in PC region. In Fig. 8 a, we have shown a scatter plot of modules having low *MedianRank* with module size. It can be seen from the figure that although for EC-MTG 15.38% modules have *MedianRank* less than ten, but three modules ‘red’ (*M* *e* *d* *i* *a* *n* *R* *a* *n* *k*=2), ‘honeydew1’ (*M* *e* *d* *i* *a* *n* *R* *a* *n* *k*=3), and ‘darkolivergreen’ (*M* *e* *d* *i* *a* *n* *R* *a* *n* *k*=3) are showing strong preservation characteristic. On the contrary, for region-pair EC-SFG, although the most of the modules have low *MedianRanks* value, but only two of them (purple and turquoise) have *MedianRank* less than three. Similarly we can see from Fig. 8 b that for HIP-SFG (37.5%) modules have *MedianRank* less than ten.

**Fig. 9:**
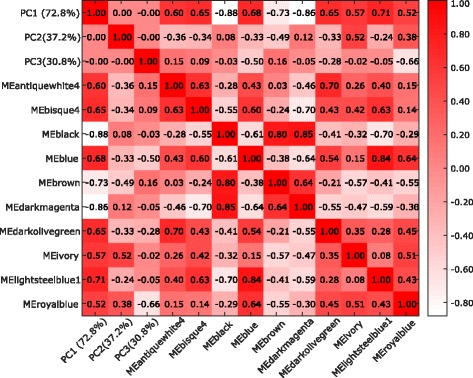
Figure shows the Heatmap of the correlation matrix formed among the eigengenes of top ranked ten modules and the first three principal components

We have performed a principal component analysis (PCA) on the expression data of DEGs in EC and HIP regions. The analysis is performed to know whether the overall expression of genes in the modules is correlated with the principal components of the DEGs expression data. We have computed the Pearson correlation among the first three principal components with the eignegenes of the identified modules in the EC region. The results are shown as a heatmap in Fig. 9 which represents the correlation between each pair of modules’ eigengenes and the first three principal components. It can be noticed that the modules showing high correlation with first principal component are also correlated with each other. For example ‘darkolivergreen’, ‘lightsteenblue1’, ‘ivory’ and ‘royalblue’ showing high correlation among their eigengenes as well as high correlation with the first principal component.

### GO and pathway analysis of preserved and non-preserved modules

To discover the biological significance of the preserved modules we have performed gene ontology (GO) and pathway based analysis. For computation convenience we have restricted our analysis for the most preserved and the most perturbed co-expressed modules. We have collected GO terms and KEGG pathways which are interrelated with the top ten ranked modules (the most preserved) and last ten ranked modules (the most perturbed) in the sorted ranked list. We have exploited the “Database for Annotation, Visualization and Integrated Discovery (DAVID)” [40] tool for performing this analysis. Table 5 shows the most significant GO terms and the significant KEGG pathway which are linked with the modules of EC-MTG regions pair. Table 4 shows the same for EC-HIP regions pair. The second column of these table shows the aggregated ranks of the modules. Column 5, 6 and 7 represents the most significant GO terms, GO identifiers and the associated *p*-Value, respectively. Column 8 and 9 shows the associated pathways and corresponding *p*-value. It can be seen from Table 5 that the most of the modules are enriched with some pathways of neuro-degenerative disorders like ‘Parkinson’s disease’ and ‘Alzheimer’s disease’.

**Table 5:** Significant GO Terms and KEGG Pathways for EC-MTG regions pair

Sl No	Module name	Aggregated rank	Module size	GO term	GO ID	*p*-Value	KEGG pathway	*p*-Value
1	Coral1	5	35	Positive regulation of dendrite extension	GO:1903861	3.10E-02	Not found	NA
2	Darkolivegreen	6	55	Cytoskeleton-dependent intracellular transport	GO:0030705	5.60E-04	Alzheimer’s disease	9.20E-05
3	Ivory	4	49	Not found	NA	NA	Not found	NA
4	Lightcyan	3	76	Regulation of translational initiation	GO:0006446	6.00E-03	GnRH signaling pathway	2.80E-02
5	Mediumorchid	8	32	Cytoskeleton organization	GO:0007010	1.50E-03	Dorso-ventral axis formation	3.50E-02
6	Navajowhite2	2	38	Response to peptide hormone	GO:0043434	8.10E-02	Not found	NA
7	Paleturquoise	7	58	Positive regulation by host of viral transcription	GO:0043923	3.30E-02	Glyoxylate and dicarboxylate metabolism	6.10E-02
8	Plum1	1	53	Dentate gyrus development	GO:0021542	3.40E-02	Not found	NA
9	Purple	10	99	Double-strand break repair via nonhomologous end joining	GO:0006303	1.50E-04	Endocrine and other factor-regulated calcium reabsorption	2.50E-03
10	Salmon	9	85	Glycolytic process	GO:0006096	8.90E-05	Metabolic pathways	1.90E-04
11	Antiquewhite4	57	34	ion transmembrane transport	GO:0034220	3.90E-02	Alzheimer’s disease	4.20E-03
12	Blue	60	137	Protein transport	GO:0015031	1.30E-02	Peroxisome	5.90E-02
13	Brown4	65	46	Cilium morphogenesis	GO:0060271	2.60E-03	Not found	NA
14	Cyan	63	80	Regulation of meiotic nuclear division	GO:0040020	1.70E-02	Ras signaling pathway	4.20E-02
15	Darkgrey	61	66	Pyruvate biosynthetic process	GO:0042866	1.60E-02	Biosynthesis of amino acids	2.50E-02
16	Darkmagenta	59	55	Antigen processing and presentation of exogenous peptide antigen via MHC class I: TAP-dependent	GO:0002479	8.00E-03	Parkinson’s disease	3.20E-02
17	Darkseagreen4	62	35	Glomerulus development	GO:0032835	1.30E-02	SNARE interactions in vesicular transport	7.60E-02
18	Greenyellow	64	94	Regulation of synaptic vesicle recycling	GO:1903421	1.20E-02	Alzheimer’s disease	3.20E-02
19	Lightsteelblue1	58	52	Positive regulation of gene expression	GO:0010628	2.60E-02	Calcium signaling pathway	1.20E-02
20	Yellow	56	130	Protein localization to plasma membrane raft	GO:0044860	1.20E-02	Endocytosis	1.70E-02

**Fig. 10:**
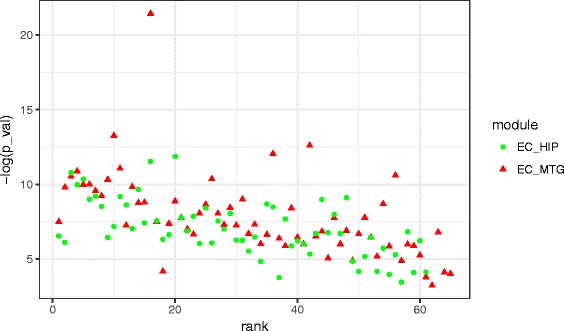
Figure shows the scatter plot between the − log(*p*-*value*) and aggregated ranks of identified modules in region pairs EC-HIP and EC-MTG. Lower *p*-value indicates higher value of − log(*p*-*value*)

It can be noted that for EC-MTG region pair, pathway enrichment is not found in four modules (module ‘coral1’, ‘ivory’, ‘navajowhite2’, and ‘brown4’) among the top ten aggregated ranked modules. However, for EC-HIP region pair top ranked modules are more enriched with pathway of neuro-degenerative disorder than the last ranked modules, shown in Table 4. It can be also noted that *p*-value associated with the GO-terms and pathways are less for top ranked modules than the 10 bottom ranked modules. Thus, the following analysis have been performed to investigate whether the aggregated ranks are incompatible with the functional enrichment. We have collected the *p*-values of GO enrichment for all the modules of EC-HIP and EC-MTG and plot those with aggregated ranks. In Fig. 10 the scatter diagram exhibits the association between *p*-value and the aggregated ranks of modules. It can be seen from the figure that top ranked modules have *p*-value lower than the bottom ranked modules.

### Analysis of preservation using ranking of modules

**Fig. 11:**
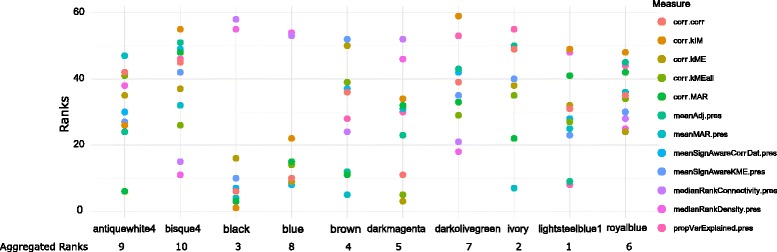
Figure shows ranking results of top ten ranked co-expression modules.The modules are ranked using 12 measures shown in the right pane of the figure

**Fig. 12:**
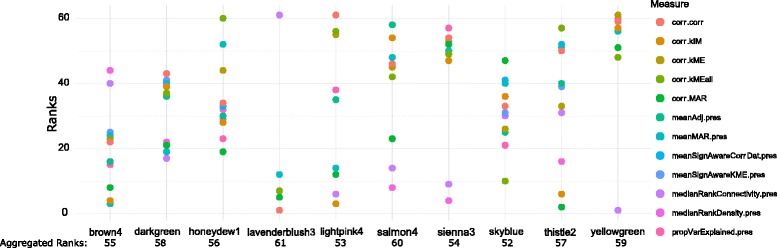
Figure shows ranking results of last ten ranked co-expression modules having ranks higher than 51. The modules are ranked using 12 measures shown in the right pane of the figure

We have compared the values of composite preservation statistics *Z*_*summary*_ and *MedianRank* for analyzing preservation pattern of co-expressed modules obtained from each pair of brain regions taking EC or HIP as references. Here, strong preservation of modules is assumed by taking *Z*_*summary*_ value greater than 10 or *MedianRank* value less than 10. Thus, the higher value of *Z*_*summary*_ or lower value of *MedianRanks* are not prioritize here, instead all the modules having *Z*_*summary*_ (or *MedianRank*) value greater than (or less than) some threshold are put into same class. So, this analysis gives the overall preservation of all modules for all pairs of regions. Thus, to analyze the preservation in modular level, here, we have proposed a rank aggregation based method which uses all preservation measures for detecting preserved modules. Here, each module receives a rank for each preservation measure. So, all the modules for a regions pair get ranks corresponding to all the preservation measures. By performing rank aggregation we aggregated all the ranks of modules to obtain a optimal rank list. Modules getting lower rank have higher preservation characteristics and vice-versa. For ranking of modules we have used the 12 preservation measures which were described in Table 3. In Figs. 11 and 12, we have shown the ranking results of some co-expression modules for EC-HIP regions pair. In Fig. 11 the ranking result of the modules having aggregated ranks less than ten are shown. Similarly, we have also shown the ranking results of co-expression modules having aggregated ranks greater than 51 in Fig. 12.

**Fig. 13:**
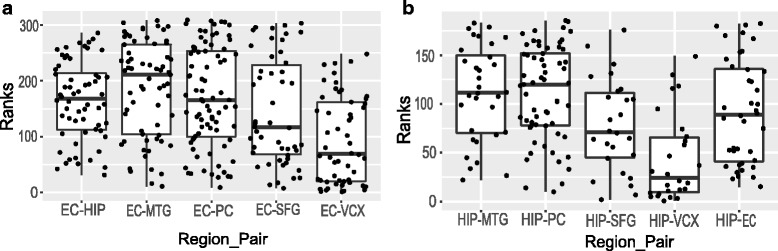
Figure shows the box and jitter plots of the aggregated ranks of all modules identified in EC (provided in panel-**a**) and HIP region (provided in panel-**b**)

To have a overall look into the preservation patterns of modules in each pair of regions, we have compared aggregated ranks. For this, we have taken all the identified modules in each pair of regions at a time, and assign ranks to them using the 12 module preservation statistics mentioned in Table 3. To make an optimal list of ranks, we have aggregated all the individual ranks similar to the process described above. In Fig. 13, we have shown the box and jitter plots of the aggregated ranks for EC (panel -a) and HIP (panel-b) regions, separately. Taking EC as reference, total 309 modules are ranked, while taking HIP as reference 185 modules are ranked. It is clear from the Fig. 13 that modules of regions VCX and SFG taking EC as reference region, have aggregated ranks lower than the other regions. It can be also noted from this figure that the modules of VCX and SFG regions get lower aggregated ranks while taking HIP as a reference region.

### Topological insights into the preserved and perturbed modules

**Fig. 14:**
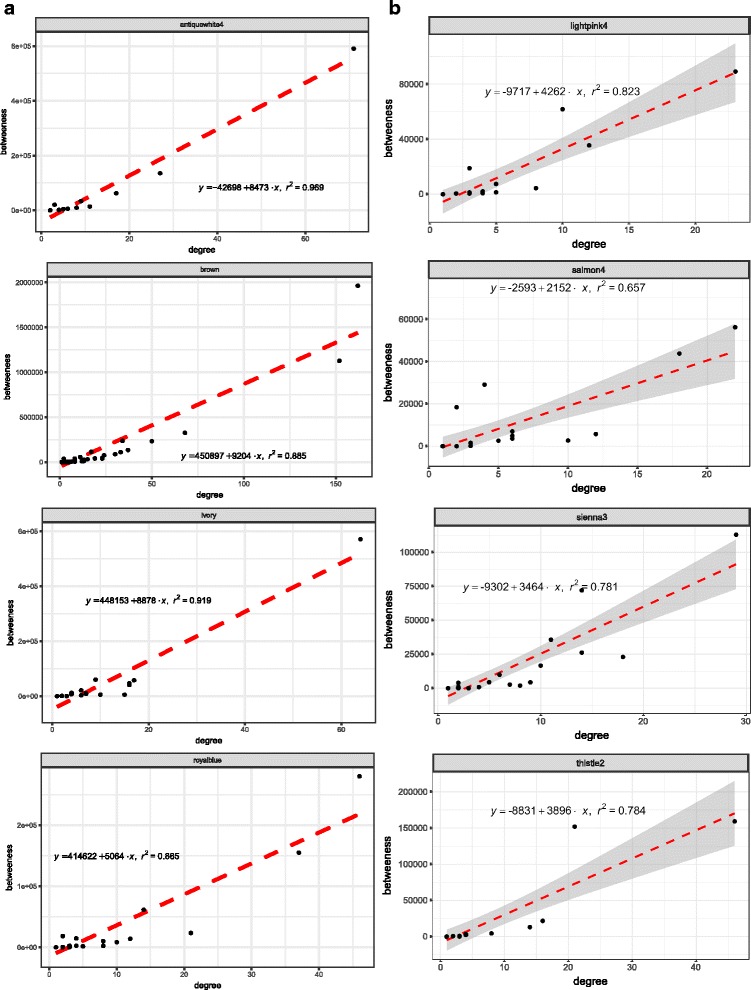
Figure shows the scatter plots of the “betweenness centrality” vs the “degree” of (**a**) 4–preserved and (**b**) 4–non-preserved modules for EC-HIP region

The following experiment have been carried out to investigate whether there exists any topological characteristics that distinguishes preserved modules from the non preserved ones. We have computed the “betweenness centrality” and the “degree” of all the proteins belonging to each preserved and non preserved module. Degree and betweenness centrality serve as important topological property of a protein in a network [41]. High degree proteins are generally called ‘hub’ whereas proteins with high betweenness centrality are called ‘bottlenecks’. Among the top ten and last ten ranked modules, four modules are selected in each category based on the higher correlation score among the betweenness centrality and the degree of their constituent proteins. Figure 14 shows scatter plots between these two metric of the selected four modules of preserved and non-preserved category. From the figure, a clear correlation pattern can be seen in preserved modules. For non preserved modules though the correlation exists but not prominent as for preserved one.

## Conclusions

In this article, we have extensively studied the preservation patterns of co-expression networks for the six distinct brain regions affected by Alzheimer’s disease (AD). For every brain region “differentially expressed genes” (DEGs) were computed using the AD affected microarray gene expression data. We have obtained the common DE genes for each pair of regions and constructed a pair of co-expression networks. The networks are then compared by using preservation statistics first introduced in [27]. The networks are partitioned into co-expression modules and these are then compared with the preservation measures. Twelve density and connectivity based measures are used here to detect preservation pattern between co-expression modules belonging to a pair of brain regions. We have also assigned ranks to each module based on the preservation measures and adopted a rank aggregation technique for combining those ranks to obtain an aggregated rank list. Low ranks of a module characterizes high preservation characteristics and vice-versa.

The whole analysis is carried out for all pairs of brain regions taking expression data of EC and HIP regions as reference. It emerges from the results of module preservation statistics (*Z*_*summary*_ value) that number of strongly preserved module for EC-MTG and HIP-MTG regions are more than other pairs of regions. Moreover, for HIP-SFG and HIP-VCX all the modules are either moderately preserved (*Z*_*summary*_ value between 2 to 10) or strongly preserved (*Z*_*summary*_ value less than 2). By considering the *MedianRank* value, modules of EC-SFG region achieves more preservation than other pairs of regions. However, for EC-MTG regions pair more number of modules has *MedianRank* value less than or equals to three. From ranking results we also got preserved and non-preserved modules for each pair of regions. A significant association among the betweenness centrality and the degree of the proteins in preserved modules have been observed from the topological analysis of the preserved and non-preserved modules. For example, in EC-HIP region, preserved modules ‘antiquewhite4’, ‘ivory’, ‘brown’ and ‘royalblue’ show a firm association among the betweenness centrality and the degree of the proteins. On the other hand for non-preserved modules like ‘thistle’, ‘sienna3’ and ‘salmon4’ the correlation is not so prominent. It reveals that the proteins belonging to the preserved modules are more prone to act as a ‘hub’ as well as ‘bottleneck’ within the whole human PPI network.

Further analysis on the preserved and non-preserved modules may facilitate to discover the exact progression pattern of the Alzheimer’s disease. Comparing expression data of six brain regions through different multivariate analysis such as MANOVA may provide useful information to the preservation structure of the modules. Detailed analysis of the expression data in all six brain regions using MANOVA may yield new insights into the preservation pattern of the brain regions. Apart from this, to know whether the genes within the top ranked modules are indeed involved with Alzheimer’s disease one can perform some experimental validation. For example one can choose to knockdown those genes to investigate whether the particular genes are really involved in Alzheimer’s disease. A proper investigation of the preserved modules of a pair of regions will yield some new insights into the development of new therapeutics for Alzheimer’s disease.

## Additional file

Additional file 1 Figure: Bar plot showing *Z*_*density*_, *Z*_*connectivity*_ and *Z*_*summary*_ values of co-expression modules having *Z*_*summary*_ greater than ten. Panel (a) shows the results for modules in EC region and panel (b) shows the same for MTG region. Both results are calculated by taking HIP region as reference. (EPS 110 kb)

## Abbreviations

DEG: Differentially expressed genes
EC: Entorhinal cortex
GEO: Gene expression omnibus
GO: Gene ontology
HIP: Hippocampus
MTG: Middle temporal gyrus
PC: Posterior cingulate cortex
PCA: Principal component analysis
SFG: Superior frontal gyrus
TOM: Topological overlap matrix
VCX: Primary visual cortex
WGCNA: Weighted gene co-expression network analysis
